# Evidence for the Efficacy of a High Dose of Vitamin D on the Hyperinflammation State in Moderate-to-Severe COVID-19 Patients: A Randomized Clinical Trial

**DOI:** 10.3390/medicina58101358

**Published:** 2022-09-27

**Authors:** Neven Sarhan, Ahmed E. Abou Warda, Rania M. Sarhan, Marian S. Boshra, Gomaa Mostafa-Hedeab, Bashayer F. ALruwaili, Haytham Soliman Ghareeb Ibrahim, Mona F. Schaalan, Shaimaa Fathy

**Affiliations:** 1 Clinical Pharmacy Department, Faculty of Pharmacy, Misr International University, Cairo 11828, Egypt; 2 Clinical Pharmacy Department, Faculty of Pharmacy, October 6 University, P.O. Box 12585, Giza 12585, Egypt; 3 Clinical Pharmacy Department, Faculty of Pharmacy, Beni-Suef University, P.O. Box 62514, Beni-Suef 62511, Egypt; 4 Pharmacology Department, Medical College, Jouf University, Sakaka 72388, Saudi Arabia; 5 Community and Family Medicine Department, Division of Family Medicine, Medical College, Jouf University, Sakaka 72388, Saudi Arabia; 6 Cardiology Department, Faculty of Medicine, El-Fayoum University, P.O. Box 63514, El Fayoum 63511, Egypt

**Keywords:** COVID-19, vitamin D, cytokine storm, inflammatory phase, low dose, high dose

## Abstract

*Background and Objectives:* Vitamin D supplementation plays a key effect in lowering cytokine storms among COVID-19 patients by influencing the activity of the renin-angiotensin system and the production of the angiotensin-2 converting enzyme. The study was conducted to explore the effect of high-dose intramuscular vitamin D in hospitalized adults infected with moderate-to-severe SARS-CoV-2 in comparison with the standard of care in the COVID-19 protocol. *Materials and Methods:* Two groups of patients were compared in this prospective randomized controlled trial as the vitamin D was administered orally to group 1 (alfacalcidol 1 mcg/day) and intramuscularly to group 2 (cholecalciferol 200,000 IU). One hundred and sixteen participants were recruited in total, with fifty-eight patients in each group. Following the Egyptian Ministry of Health’s policy for COVID-19 management, all patients received the same treatment for a minimum of five days. *Results:* A significant difference was recorded in the length of hospital stay (8.6 versus 6.8 days), need for high oxygen or non-invasive mechanical ventilator (67% versus 33%), need for a mechanical ventilator (25% versus 75%), clinical improvement (45% versus 55%), the occurrence of sepsis (35% versus 65%), and in the monitored laboratory parameters in favor of high-dose vitamin D. Moreover, clinical improvement was significantly associated with the need for low/high oxygen, an invasive/non-invasive mechanical ventilator (MV/NIMV), and diabetes, while mortality was associated with the need for MV, ICU admission, atrial fibrillation, chronic obstructive pulmonary disease, asthma, and the occurrence of secondary infection. *Conclusions:* Our study showed that high-dose vitamin D was considered a promising treatment in the suppression of cytokine storms among COVID-19 patients and was associated with better clinical improvement and fewer adverse outcomes compared to low-dose vitamin D.

## 1. Introduction

By the year 2020, the severe acute respiratory syndrome coronavirus-2 (SARS-CoV-2) infection reached epidemic levels and was responsible for the deaths of over 4 million people around the world [1]. Therefore, the COVID-19 outbreak has prompted a global effort to combat the disease and identify risk factors and prognosis markers [2]. Vitamin D insufficiency is an example of the factors which may affect snowballed COVID-19 risk and mortality [3].

As a global public health issue, vitamin D deficiency is also considered an epidemic in Europe and the United States. It is associated with a rise in infectious and non-infectious conditions, particularly upper respiratory tract infection [4]. Several health organizations of different governments have recommended that vitamin D supplements are to be taken during the summer months of the COVID-19 pandemic as people are strongly encouraged to spend as much time as they can at home [5].

Vitamin D functions as a secosteroid hormone that is soluble in fat [6,7]. It is also known as cholecalciferol (vitamin D3) or ergocalciferol (vitamin D2), which are hormone precursors and play a crucial role in regulating calcium and phosphate metabolism [8]. Moreover, it was found that vitamin D has an anti-inflammatory effect and is associated with a significantly lower level of inflammatory mediators including cytokines, tumor necrosis factors, and interleukins [9].

In addition, a recent study demonstrated that vitamin D is essential for immunological function and cellular resilience, and its absence may lead to cytokine storms in new coronavirus infections [10]. Therefore, it can prevent acute respiratory distress syndrome (ARDS) by decreasing the synthesis of pro-inflammatory Th1 cytokines, including tumor necrosis factor-alpha (TNF) and interferon, while simultaneously boosting anti-inflammatory cytokine expression by macrophages [9]. The contribution of vitamin D to the integrity of the physical barriers that keep viruses from entering body tissues and causing infection has been also reported [11].

**Figure 1:**
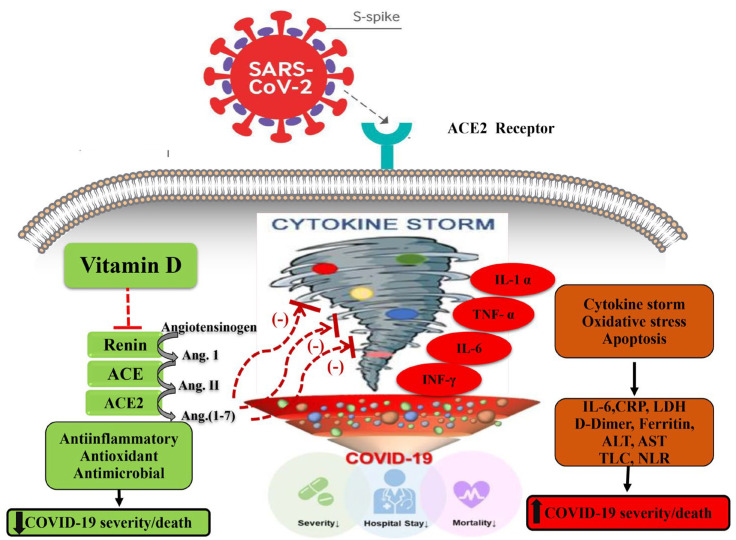
Potential mechanisms by which high-serum vitamin D levels may confer protective effects against a COVID-19–induced inflammatory state and increased severity and/or mortality. ACE, angiotensin-converting enzyme; ACE 2, angiotensin-converting enzyme 2, COVID-19, coronavirus disease 2019. Arrows pointing up indicate an increase; arrows pointing down indicate a decrease; red dashed lines indicate inhibition.

In addition, the adverse outcomes of COVID-19 may be circumvented by vitamin D by modulating the activity of the renin-angiotensin system (RAS) and the expression of the angiotensin-2 converting enzyme (ACE2), which reduces pulmonary permeability in the ARDS experimental model. ACE2 either activates or represses the expression of various genes by binding to the vitamin D response elements (VDRE) found in the gene promoter region (Figure 1) [12,13]. This step is critical because SARS CoV-2 has been shown to infect host cells via employing ACE2 as a receptor [14] and downregulates the expression of ACE2 [15,16]. The downregulation of ACE2 throughout COVID-19 infection causes a cytokine storm in the host, resulting in ARDS [17,18].

In light of the benefit, low risk, and cost of vitamin D therapy in COVID-19, it has been suggested in numerous recent studies that the dosages of vitamin D should be raised [19,20].

Following this recommendation, the present study was quested to determine whether the clinical results and prognoses of COVID-19 patients are enhanced by supplementing a high dose of vitamin D relative to the conventional low dose.

## 2. Methods

### 2.1. Study Design and Patients

From December 2020 to June 2021, a total of one hundred and sixteen adults, 18 years of age or older, with a verified COVID-19 hyperinflammation status were recruited at Teacher’s Hospital, Cairo, Egypt.

The Beni-Suef University Research Ethics Committee approved their participation in an off-label therapeutic program (REC-H-PhBSU-21017), and the clinical trials registry recorded their enrollment (ClinicalTrials.gov NCT04738760). The study was conducted in accordance with the Helsinki Declaration and in accordance with amended good clinical practices [21]. All participants or their legal guardians provided written informed consent.

**Figure 2:**
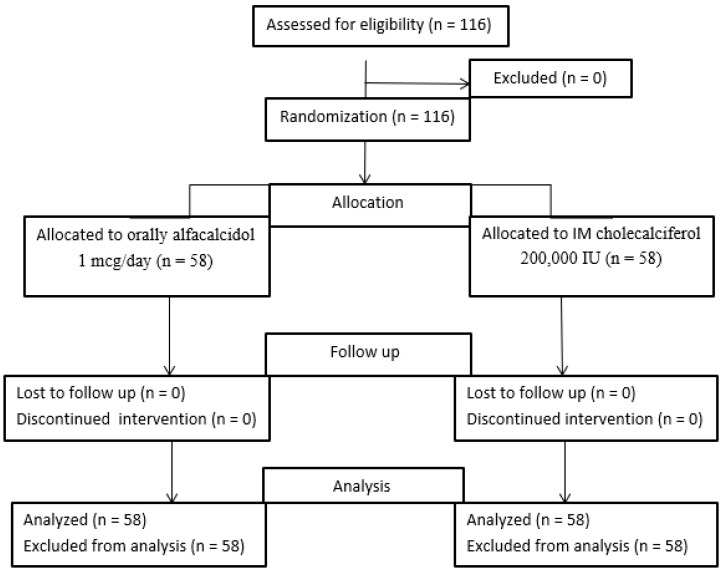
Patients flow chart.

The current study was a prospective randomized controlled study in which patients were randomly allocated in a 1:1 ratio into two groups (Figure 2).

Group 1 included SARS-CoV-2-infected patients and received treatment with a standard dose of vitamin D alfacalcidol (1 microgram/day) orally as a standard of care in COVID-19 management.

Group 2 included moderate and severe SARS-CoV-2-infected patients and received treatment with a single high-dose vitamin D cholecalciferol (200,000 IU) IM in addition to standard COVID-19 management.

Simple randomization was carried out by allocating patients using a table of random numbers.

### 2.2. Eligibility Criteria

Eligible patients were 18 years of age or older, hospitalized with pneumonia verified by chest CT scan, and had a positive COVID-19 RT-PCR (COVID-19 IgG/IgM Rapid Test Kit, Abbexa, Cambridge, UK) result. The strain characterized in our study is the D 614G mutant strain, called the Wuhan alpha strain, which was prevalent in Egypt in late 2020. The hyperinflammation was determined by a rise in lactate dehydrogenase (LDH, >220 U/L), ferritin (900 ng/mL, normal value 400 ng/mL), or C-reactive protein (CRP, ≥ 100 mg/L, normal values < 6 mg/L), and at least one of the following factors needed to have occurred: oxygen saturation ≤ 93 percent on room air, respiratory frequency ≥ 30/min, PaO_2_/FiO_2_ (arterial oxygen partial pressure/inspired oxygen fraction) = 300 lung involvement worsening, described as a rise in the number and/or extension of the areas of pulmonary consolidation, need for increased FiO_2_ to keep the stability of O_2_ saturation, or worsening in O_2_ saturation if more than 3% with stable FiO_2_.

In the prior month, patients who took vitamin D supplementation and those with contraindications for vitamin D supplementation were excluded. These contraindications included active granulomatosis (tuberculosis, sarcoidosis, and lymphoma), calcic lithiasis history, confirmed hypervitaminosis D or hypercalcemia, and intolerance to vitamin D. In addition, exclusion criteria included pregnancy or the requirement to be admitted to a resuscitation or intensive care unit due to organ failure.

### 2.3. Treatment

All patients received the following standard treatment for at least five days: 400 mg hydroxychloroquine once per day, 400/100 mg lopinavir/ritonavir twice per day, or 200 mg remdesivir loading dose then 100 mg as a maintenance dose once daily and 6 mg dexamethasone once daily. Moreover, the anticoagulant enoxaparin as a prophylactic was subcutaneously injected once per day if the D-dimer was from 500 to 1000, or enoxaparin as a treatment was subcutaneously injected twice per day if the D-dimer was >1000. Additionally, all patients received 1 gm paracetamol every 6 h and 25 mg quetiapine once daily at bedtime as a supportive treatment.

### 2.4. Study Outcomes

First, patients’ clinical improvement was assessed as a primary outcome, defined as an improvement of oxygenation parameters. Secondary outcomes included hospital stay length, mortality, variation in inflammatory markers levels, C-reactive protein (CRP), lactate dehydrogenase (LDH), ferritin, and occurrence of secondary infections including bacterial or fungal infections as well as the occurrence of at least one adverse event.

### 2.5. Sample Size Calculation

According to our power calculation, a sample size of 116 patients provided us with 80% power to detect an effect size f2 of 0.15, assuming a two-sided tail hypothesis and an alpha level = 0.05 [22,23].

### 2.6. Biochemical Analysis

First, 5 mL of peripheral venous blood in a plane vacutainer tube was withdrawn for the assessment of a complete blood count (CBC) (using Sysmex XT-1800i, Kobe, Japan), liver functions, alanine transaminase (ALT), and aspartate aminotransferase (AST), using spectrophotometric assay on Advia chemistry 2400 XPT, Siemens Healthineers (Erlangen, Germany), automated quantitative C-reactive protein (CRP), and lactate dehydrogenase (LDH) (using Roche Diagnostics, Mannheim, Germany).

Then, 5 mL of peripheral venous blood was obtained in a vacutainer tube containing citrate for the assessment of an automated quantitative D-Dimer using D-Dimer Reagent Specification commercially available by HEALES; Shenzhen Housing Technology; China.

The enzyme-linked immunosorbent assay (ELISA) is an effective method for measuring cytokines. The Human IL-6 ELISA kit tested serum IL-6 (Shanghai Sunred Biological Technology Co., Ltd., Shanghai, China). Serum ferritin and troponin were measured using the Human Ferritin ELISA kit and a Human Troponin ELISA kit (eBioscience, San Diego, CA, USA). The manufacturer’s ELISA steps were followed.

### 2.7. Statistical Analysis

The statistical Package for the Social Sciences (SPSS) version 22.0 was used in the analysis of the data (SPSS, Chicago, IL, USA). The mean and standard deviation were used to present continuous data. The presentation of categorical data was as numbers and percentages.

Using the Kolmogorov–Smirnov and Shapiro–Wilk tests, the data’s normality was examined. The Student’s *t*-test was utilized to compare normally distributed numeric variables between the two groups. Using ANOVA, comparisons between groups and changes over time were made. The Chi-square test was employed to analyze categorical data. The Mann–Whitney test was utilized to compare non-normally distributed data between groups. Using the Wilcoxon signed rank test, comparisons were made between two numerical measurements across time. Regarding categorical data, Fisher’s exact tests were utilized to compare the groups.

The contribution of biochemical and clinical predictors to each outcome of ©nterest was determined by regression analysis. In a univariate analysis, we included clinical covariates and additional variables linked with the outcome at a *p*-value < 0.2. The final model contained clinical predictors with a significance level of *p*-value < 0.05. The Kaplan–Meier and log-rank tests were employed to compare survival analyses. All *p*-values were two-sided, and a value < 0.05 was statistically significant.

## 3. Results

### 3.1. Baseline Patients’ Characteristics

Of the total of 116 hospitalized COVID-19 patients who were included in the study, 58 patients received treatment with low-dose vitamin D and 58 received treatment with high-dose vitamin D. Baseline characteristics of the two groups are presented in Table 1. Mean age, inflammatory mediators (lactate dehydrogenase (LDH), serum ferritin, C-reactive protein (CRP), liver enzymes, oxygen saturation, and respiratory rate) were similar in both groups with no statistically significant difference.

### 3.2. Changes in Biochemical Parameters between Both Groups

**Figure 3:**
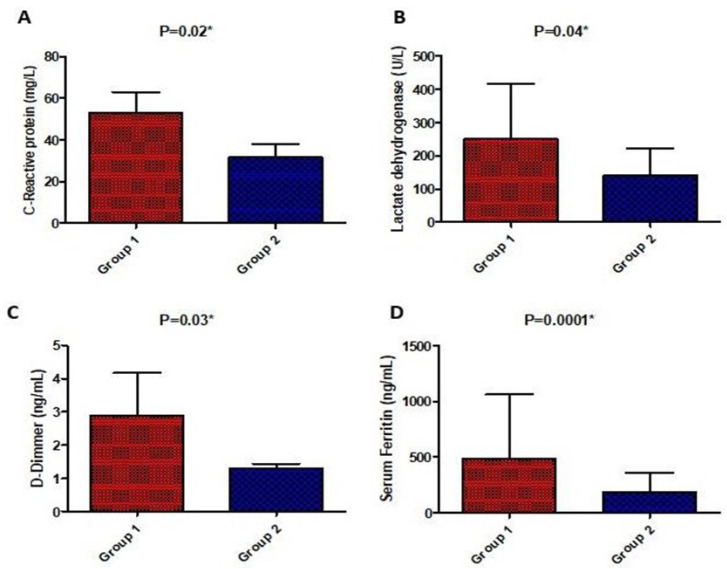
Comparison of serum biochemical parameters (C-reactive protein (**A**), lactate dehydrogenase (**B**), D-Dimer (**C**), and ferritin (**D**)) post-treatment between the studied groups. Values are illustrated as means ± SD. * represents a significant difference between both groups at *p* < 0.05.

**Figure 4:**
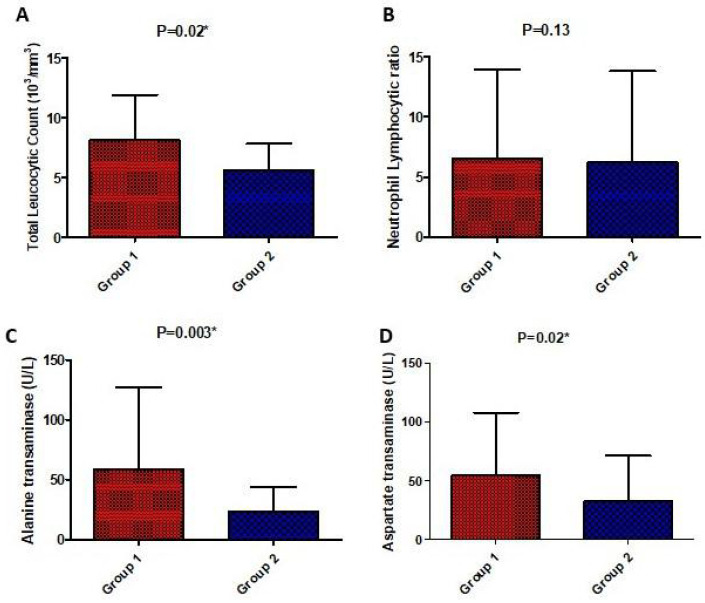
Comparison of laboratory parameters (total leucocytic count (**A**), neutrophil lymphocytic ratio (**B**), serum alanine transaminase (**C**), and aspartate transaminase (**D**)) post-treatment between the studied groups. Values are illustrated as means ± SD. * represents a significant difference between both groups at *p* < 0.05.

There was a significant difference in laboratory parameters between both groups in favor of the high-dose vitamin D group including CRP, LDH, D-Dimer, ferritin, TLC, AST, and ALT which were significantly lower in the high-dose vitamin D group compared to the low-dose vitamin D group, except for the respiratory rate and neutrophils lymphocyte ratio which were not significantly different. On the other hand, post-treatment, liver enzymes were significantly higher in the tocilizumab/infliximab group as shown in Figure 3 and Figure 4.

### 3.3. Impact of Both Treatment Arms on the Clinical Outcomes

A significant difference was recorded between the two groups in the primary outcomes including clinical improvements in terms of the need for non-invasive mechanical ventilator/high oxygen, oxygen saturation, respiratory rate, and P/F ratio as well as the secondary outcomes including ICU admission, length of hospital stay, improvement time, and occurrence of sepsis, as presented in Table 2. By the end of treatment, 55% of patients in the high-dose vitamin D group (group 2) compared to 45% of patients in the low-dose vitamin D (group 1) were severe in illness (*p* = 0.09). In group 2, 47% of the patients compared to 51% of the patients in group 1 needed low oxygen (*p* = 0.42). In group two, 67% of the patients in compared to 33% of the patients in group 1 needed high oxygen or a non-invasive mechanical ventilator (NIV) (*p* = 0.03). In group 2, 25% of the patients compared to 67% of the patients in group 1 needed an invasive mechanical ventilator (MV) (*p* = 0.03). In group 2, 42% of the patients compared to 65% of the patients in group 1 required ICU admission (*p* = 0.016). In group 2, 55% of the patients compared to 29% of the patients in group 1 showed clinical improvement (*p* = 0.03). In group 2, 45% of the patients compared to 55% of the patients in group 1 died (*p* = 0.49). In group 2, 33% of the patients compared to 64% of the patients in group 1 showed secondary bacterial infection in the form of sepsis (*p* = 0.04).

### 3.4. Predictors of Clinical Improvement by Binary Logistic Regression Analysis

The binary logistic regression analysis indicated that better clinical improvement and less severe COVID-19 symptoms were associated with the need for low oxygen (OR = 6.67, C.I. = 2.07–21.35, *p* = 0.001) and inversely associated with the need for high oxygen and NIMV (OR = 6.19, C.I. = 0.41–0.86, *p* = 0.031), the need for an invasive mechanical ventilator (MV) (OR = 0.83, C.I. = 0.64–0.98, *p* = 0.012), diabetes (OR = 0.37, C.I. = 0.33–0.56, *p* = 0.045), atrial fibrillation (OR = 0.41, C.I.= 0.15–0.38, *p* = 0.008), and the occurrence of secondary infection (OR = 0.33, C.I. = 0.16–0.94, *p* = 0.004), as shown in Table 3.

However, after conducting a multiple logistic regression with covariates reporting a *p*-value less than 0.2 in the univariate regression analysis, only the need for low oxygen (adjusted OR = 5.53, C.I. = 2.41–15.12, *p* = 0.005), the need for high oxygen/NIMV (adjusted OR = 0.23, C.I. = 0.31–0.78, *p* = 0.041), the need for invasive MV (adjusted OR= 0.71, C.I. = 0.58–0.81, *p* = 0.038), diabetes (adjusted OR = 0.42, C.I. = 0.28–0.74, *p* = 0.048), and the occurrence of secondary bacterial infection (adjusted OR = 0.46, C.I.= 0.31–0.83, *p* = 0.02) remained significant, as shown in Table 4.

### 3.5. Predictors of Mortality by Binary Logistic Regression Analysis

The binary logistic regression analysis revealed that COVID-19 mortality was associated with the need for MV (OR = 4.93, C.I. = 1.1–12.56, *p* = 0.039), ICU admission (OR = 4.58, C.I. = 2.33–9.51, *p* = 0.009), atrial fibrillation (OR = 2.39, C.I. = 1.7–2.3, *p* = 0.03), chronic obstructive pulmonary disease (COPD) (OR = 1.93, C.I. = 1.7–7.4, *p* = 0.015), asthma (OR = 3.07, C.I. = 2.4–8.9, *p* = 0.023), and the occurrence of secondary infection (OR = 7.2, C.I. = 6.73–13.6, *p* = 0.0003), as shown in Table 4.

**Table 4:** Predictors of mortality by binary logistic regression analysis.

Risk Factor	Odd Ratio	95%CI	*p*-Value
Need for low oxygen	0.16	0.05–0.53	0.002 *
Need for high oxygen/NIMV	0.19	0.025–1.45	0.11
Need for invasive MV	4.93	1.1–12.56	0.039 *
ICU admission	4.58	2.33–9.51	0.009 *
Hypertension	1.42	0.15–1.21	0.11
Diabetes	1.37	0.33–5.56	0.45
Heart failure	1.42	0.14–2.31	0.42
Chronic kidney disease	0.63	0.31–5.45	0.71
Chronic liver disease	1.49	0.03–1.82	0.86
Ischemic heart disease	2.07	0.44– 9.76	0.35
Atrial fibrillation	2.39	1.7– 2.3	0.03 *
Chronic obstructive pulmonary disease	1.93	1.7–7.4	0.015 *
Asthma	3.07	2.4–8.9	0.023 *
Occurrence of secondary infection	7.2	6.73–13.6	0.0003 *
Vitamin D dose (high/low)	0.73	0.63–0.83	0.002 *

NIMV; non-invasive mechanical ventilator, MV; mechanical ventilator, ICU; intensive care unit, *: significant, level of significance <0.05.

However, after conducting a multiple logistic regression with covariates reporting a *p*-value less than 0.2 in the univariate regression analysis, only the need for invasive MV (adjusted OR = 5.01, C.I. = 3.6–10.9, *p* = 0.002), ICU admission (adjusted OR = 6.4, C.I. = 3.71–11.4, *p* = 0.009), atrial fibrillation (adjusted OR = 3.1, C.I.= 2.09–4.2, *p* = 0.041), COPD (adjusted OR = 1.82, C.I. = 1.3–8.9, *p* = 0.03), and the occurrence of secondary bacterial infection (adjusted OR = 3.3, C.I. = 2.8–11.6, *p* = 0.009) remained significant, as shown in Table 5.

## 4. Discussion

Although it is impossible to compare global COVID-19 numbers, it is evident that the death rate is significant in many nations. Several variables, including age, the quality of the healthcare system, overall health, and socioeconomic level, are related to bad results [24].

Vitamin D levels and vitamin D therapy may be underestimated determinants in COVID-19 treatment. However, the benefits of supplementing COVID-19 patients with vitamin D remain debatable [25]

This is the first trial to compare treatment with high-dosage vitamin D versus treatment with low-dose vitamin D in patients with moderate-to-severe COVID-19.

Deficiency in vitamin D is shown to be linked to lung inflammation exacerbation, resulting in ARDS along with the destruction of respiratory epithelium and hypoxia [7]. In critically ill patients, 25-OH-cholecalciferol levels are inversely linked with the incidence of abrupt respiratory failure. In addition, Biesalski et al. (2020) emphasized that there is a deadly association between vitamin D deficiency and comorbidities in COVID-19 patients [26]. In addition, another study indicated that optimizing the vitamin D status certainly has benefits in COVID-19 patients [27]. This study revealed that the incidence of mechanical ventilation, ICU hospitalization, death, sepsis, and atrial fibrillation in the high-dosage vitamin D group was considerably reduced compared to the low-dosage vitamin D group. However, the need for high oxygen was significantly higher in the high-dose vitamin D group compared to the low-dose group. Additionally, there was a significant difference in the monitored parameters before and after treatment in favor of the high-dose vitamin D group including CRP, LDH, D-Dimer, ferritin, TLC, AST, and ALT, which were significantly lower in the high-dose vitamin D group compared to the low-dose group. Furthermore, a greater percentage of patients who were given large doses of vitamin D demonstrated better clinical improvement, a shorter amount of time needed for improvement, and a shorter amount of time spent in the hospital.

It was found by Tan et al. (2020) that taking vitamin D supplements was linked to a remarkable decrease in the proportion of older COVID-19 patients who needed oxygenation and/or critical care support due to clinical deterioration. This finding is very similar to what was found in the current study [28]. In addition, Castillo and colleagues demonstrated that the administration of vitamin D, as opposed to a placebo, decreased the number of COVID-19 patients who required intensive care unit treatment in 76 hospitalized patients who were receiving the most effective treatment available [29].

According to the findings of another observational study, treatment with vitamin D as a booster medication appeared to be associated with a lower risk of mortality in acute in-patients who were being treated for COVID-19. This was the case regardless of the baseline serum 25(OH)D levels of the patients [30]. Moreover, a number of studies indicate that the severity of hypovitaminosis D appears to be associated with a poor prognosis of COVID-19. This is because COVID-19 cases that are accompanied by hypovitaminosis D are more likely to exhibit severe COVID-19 symptoms, which can lead to death [7,31]. Both the mean blood concentration of vitamin D and the levels of IL-6 were revealed to be independent predictors of COVID-19 severity and death. Significant negative relationships were established between the two variables [32].

Insufficiency in vitamin D was found to be a risk factor for positive COVID-19 testing in a study that was conducted in Los Angeles. According to the findings of the study, a significantly lower percentage of patients in critical care units had vitamin D levels that were above 50 nmol/L as compared to normal levels [33]. In addition, strong associations between vitamin D deficiency and hospitalization, illness severity, and mortality, especially in the elderly, have been recently indicated by a non-peer-reviewed study conducted in Cincinnati [29]. This is owing to the fact that a severe lack of vitamin D is considered to be a reliable predictor of community-acquired pneumonia [34].

Similarly, a meta-analysis by Ghasemian et al. (2021) found that the severity of COVID-19 is linked to the lack of vitamin D, which is in line with findings from a more recent study by Anuruddhika et al. Vitamin D deficiency was observed to increase the chance of acquiring COVID-19 to 5.1 times larger than that with appropriate levels of vitamin D. SARS-CoV-2 was shown to be three times more common in patients with vitamin D deficiency, putting them at nearly five times the risk of acquiring the most severe form of the disease [35,36]. These results are in line with the research that has questioned the relationship between vitamin D and COVID-19 [37]. Additionally, the results of a recent study that was conducted on COVID-19 patients showed that older COVID-19 patients who used vitamin D3 supplements had a greater survival rate after three months [38].

In contrast, a large dose of vitamin D3 is not recommended to treat moderate to severe COVID-19, according to the findings of Muari et al. They found that compared to a placebo, a single high dose of vitamin D3 did not significantly reduce the length of stay for COVID-19 patients in the hospital [39].

The Shade study came to a conclusion that was remarkably similar to this investigation; the use of vitamin D therapy in COVID-19-infected patients was associated with a shorter recovery duration. Infection with SARS-CoV-2 transformed SARS-CoV-2 RNA negative and fibrinogen dropped dramatically with high-dose cholecalciferol supplementation [40].

Patients with vitamin D insufficiency showed significantly greater serum ferritin levels compared to those with normal vitamin D levels, according to a study by Jain et al. Patients with COVID-19 who had low levels of vitamin D also had greater levels of TNF- in their blood [41].

In addition, an independent risk factor for poor prognosis in COVID-19 patients is a lower vitamin D level at the time of hospital admission. This finding suggests that the risk of SARS-CoV-2 infection leading to hospitalization of COVID-19 patients may be increased with hypovitaminosis D [42].

Another study by Lakkireddy found that supplementing with vitamin D reduced the levels of inflammatory indicators by a significant margin following adjunct treatment pulse D therapy, as seen by analyses of inflammatory markers performed before and after the treatment in the vitamin D group. In comparison, the non-vitamin D group’s drop in inflammatory markers was insignificant. A highly significant difference was seen between the vitamin D and non-vitamin D groups in terms of lowering the inflammatory markers [41,43].

Lastly, the nutritional state has been regarded as a crucial determinant, despite its ineffectiveness in many severe viral infections due to the unsatisfactory efficacy of nutrient supplements delivered at the advanced stage of the disease. Nonetheless, it is essential to be aware that a healthy nutritional status, if acquired prior to disease progression, might boost the immune system and anti-inflammatory benefits.

Widening pre-clinical and clinical evidence supports vitamin D as a biological predictor of COVID-19 outcomes. In the absence of preventive or curative treatment, several scientific societies have not waited for interventional data to recommend supplementing adults with vitamin D to prevent the onset of COVID-19 [44,45]. Moreover, future publications are needed for assessing whether high-dose vitamin D supplementation may significantly improve the clinical presentation of COVID-19 and its prognosis, and if it plays a vital role in decreasing the mortality rate in high-risk patients or not.

## 5. Conclusions

The current study concluded that the sooner micronutrients are administered to outpatients, the better the outcome, especially before supportive or specific treatment is commenced. This is a simple, inexpensive, and beneficial strategy. Despite the fact that micronutrients in high doses may be required to restore deficits, it is strongly advised to adhere to the recommended daily intake of vitamin D over the long term to stimulate the immune system, particularly in the regulation of cytokine response against infections in the era of COVID-19. However, additional research with larger sample sizes and more stringent inclusion criteria may be required to determine the complete impact of high-dose vitamin D treatment in COVID-19 patients.

## Figures and Tables

**Table 1:** Baseline characteristics and demographic data of studied groups.

Variable	Group 1 (Low-Dose Vitamin D) n = 58	Group 2 (High-Dose Vitamin D) n = 58	Significance *p* < 0.05
**Age (mean ± SD)**	65.7 ± 12.6	66.1 ± 11.2	*p* = 0.16
**Male gender (n)**	46	38	*p* = 0.07
**Oxygenation (mean ± SD)**			
**Oxygen saturation**	88.3 ± 8.8	86.4 ± 13.4	*p* = 0.2
**P/F ratio**	197.3 ± 112.8	180.8 ± 99.7	*p* = 0.56
**Respiratory rate**	24.2 ± 4.4	25.9 ± 5.7	*p* = 0.21
**Biochemical markers (mean ± SD)**			
**Baseline C- reactive protein (CRP)**	120.5 ± 100.4	145.5 ± 101.2	*p* = 0.18
**Baseline interleukin-6 (IL-6)**	15.7 ± 2.3	17.1 ± 1.6	*p* = 0.21
**Baseline lactate dehydrogenase (LDH)**	350.1 ± 140.6	360.6 ± 136.5	*p* = 0.72
**Baseline D-Dimer**	1.39 ± 1.8	1.1 ± 1.4	*p* = 0.29
**Baseline ferritin**	886.5 ± 87.2	704.1 ± 59.3	*p* = 0.17
**Serum creatinine**	1.26 ± 0.96	1.41 ± 0.59	*p* = 0.31
**Total leucocytes count (TLC)**	7.8 ± 3.6	8.2 ± 3.9	*p* = 0.60
**Neutrophil lymphocytic ratio (NLR)**	9.8 ± 8.8	7.2 ± 3.8	*p* = 0.06
**Alanine transaminase (ALT)**	52.1 ± 6.7	41.6 ± 3.2	*p* = 0.29
**Aspartate transaminase (AST)**	52.6 ± 4.3	45.2 ± 3.1	*p* = 0.28
**Comorbidities (n)**			
**Hypertension**	31	31	*p* = 0.57
**Diabetes**	22	23	*p* = 0.6
**Heart failure**	0	2	*p* = 0.25
**Chronic kidney disease**	4	0	*p* = 0.07
**Chronic liver disease**	2	0	*p* = 25
**Ischemic heart disease**	10	10	*p* = 0.6
**Atrial fibrillation**	4	0	*p* = 0.07
**Asthma**	0	2	*p* = 0.24
**Chronic obstructive pulmonary disease**	2	2	*p* = 0.67
**Medications (n)**			
**Hydroxychloroquine**	10	12	*p* = 0.15
**Remdesivir**	50	58	*p* = 0.12
**Lopinavir/ritonavir**	15	21	*p* = 0.23
**Ivermectin**	15	13	*p* = 0.41
**Infliximab**	58	56	*p* = 0.828
**Tocilizumab**	46	50	*p* = 0.34

NIMV; non-invasive mechanical ventilator, MV; mechanical ventilator, SD; standard deviation, n; the number of cases within the group, (n); the number of patients.

**Table 2:** Comparison between the studied groups for COVID-19 clinical outcomes.

Variable	Group 1 (Low-Dose Vitamin D) n = 58	Group 2 (High-Dose Vitamin D) n = 58	Significance
**Primary Outcomes**			
**Clinical improvement (%);**			ꭓ^2^ = 4.7
No/Yes	71/29	45/55	*p* = 0.03 *
**Need for low oxygen (%);**			ꭓ^2^ = 0.16
No/Yes	49/51	53/47	*p* = 0.42
**Need for NIMV (%);**			χ^2^ = 2.21
No/Yes	67/33	33/67	*p* = 0.03 *
**Need for invasive MV (%);**			χ^2^ = 3.1
No/Yes	33/67	75/25	*p* = 0.03 *
**Oxygen saturation**	88.4 ± 8.3	95.2 ± 5.7	*p* = 0.04 *
**Respiratory rate**	16.9 ± 5.7	14.2 ± 4.4	*p* = 0.38
**P/F ratio**	321 ± 97.3	156.1 ± 58.9	*p* = 0.003 *
**Secondary Outcomes**			
**ICU admission (%);**			χ^2^ = 5.5
No/Yes	35/65	58/42	*p* = 0.016 *
**Death (%);**			χ^2^ = 1.4
No/Yes	49/51	55/45	*p* = 0.49
**Occurrence of sepsis (%);**			χ^2^ = 4.1
No/Yes	36/64	67/33	*p* = 0.04 *
**Length of hospital stay**	8.9 ± 5.1	6.1 ± 3.4	*p* = 0.04 *
**Time to improvement**	8.8 ± 4.7	6.27 ± 2.5	*p* = 0.002 *

NIMV; non-invasive mechanical ventilator, MV; mechanical ventilator, ICU; intensive care unit, %: percentage of cases within the group, χ^2^: Chi-square value, *: significant difference < 0.05.

**Table 3:** Predictors of clinical improvement by binary logistic regression analysis.

Risk Factor	Odd Ratio	95%CI	*p*-Value
Need for low oxygen	6.67	2.07–21.35	0.001 *
Need for high oxygen/NIMV	0.19	0.41–0.86	0.03 *
Need for invasive MV	0.83	0.64–0.98	0.01 *
ICU admission	0.24	0.45–11.1	0.81
Hypertension	0.55	0.91–7.14	0.074
Diabetes	0.37	0.33–0.56	0.04 *
Heart failure	0.719	0.65–1.79	0.94
Chronic kidney disease	0.63	0.31–5.45	0.71
Chronic liver disease	0.71	0.64–0.79	0.58
Ischemic heart disease	0.45	0.07–2.11	0.31
Atrial fibrillation	0.41	0.15–0.38	0.008 *
Chronic obstructive pulmonary disease	0.85	0.09–8.4	0.98
Asthma	0.30	0.04–2.5	0.45
Occurrence of secondary infection	0.33	0.16–0.94	0.004 *
Vitamin D dose (high/low)	4.3	3.2–6.9	0.001 *

NIMV; non-invasive mechanical ventilator, MV; mechanical ventilator, ICU; intensive care unit, *: significant, level of significance <0.05.

**Table 5:** Factors associated significantly with clinical improvement and mortality as determined by the multiple logistic regression analysis.

Variables	Adjusted OR	Adjusted 95%CI	Adjusted *p*-Value
**Factors associated with clinical improvement**			
Need for low oxygen	5.35	2.41–15.12	0.005 *
Need for high oxygen/NIMV	0.23	0.31–0.78	0.041 *
Need for invasive MV	0.71	0.58–0.81	0.038 *
Diabetes	0.42	0.28–0.74	0.048 *
Occurrence of secondary infection	0.46	0.31–0.83	0.02 *
Vitamin D dose (high/low)	3.9	2.4–5.7	0.03 *
**Factors associated with clinical mortality**			
Need for invasive MV	5.01	3.6–10.9	0.002 *
ICU admission	6.4	3.71–11.4	0.009 *
Atrial fibrillation	3.1	2.09–4.2	0.041 *
Chronic obstructive pulmonary disease	1.82	1.3–8.9	0.03 *
Occurrence of secondary infection	3.3	2.8–11.6	0.009 *
Vitamin D dose (high/low)	0.68	0.59–0.81	0.003 *

NIMV; non-invasive mechanical ventilator, MV; mechanical ventilator, ICU; intensive care unit, *: significant, level of significance <0.05, variables with *p*-value <0.2 were included in the model.

## Data Availability

Not applicable.

## References

[1] Sharma A., Tiwari S., Deb M.K., Marty J.L. (2020). Severe acute respiratory syndrome coronavirus-2 (SARS-CoV-2): A global pandemic and treatment strategies. Int. J. Antimicrob. Agents.

[2] Schaalan M., Warda A.E.A., Osman S.M., Fathy S., Sarhan R.M., Boshra M.S., Sarhan N., Gaber S., Ali A.M.A. (2022). The Impact of Sociodemographic, Nutritional, and Health Factors on the Incidence and Complications of COVID-19 in Egypt: A Cross-Sectional Study. Viruses.

[3] Baktash V., Hosack T., Patel N., Shah S., Kandiah P., van den Abbeele K., Mandal A.K.J., Missouris C.G. (2021). Vitamin D status and outcomes for hospitalized older patients with COVID-19. Postgrad. Med. J..

[4] De La Puente-Yagüe M., Cuadrado-Cenzual M.A., Ciudad-Cabañas M.J., Hernández-Cabria M., Collado-Yurrita L. (2018). Vitamin D: And its role in breast cancer. Kaohsiung J. Med. Sci..

[5] Pereira M., Dantas Damascena A., Galvão Azevedo L.M., de Almeida Oliveira T., da Mota Santana J. (2022). Vitamin D deficiency aggravates COVID-19: Systematic review and meta-analysis. Crit. Rev. Food Sci. Nutr..

[6] Annweiler C., Legrand E., Souberbielle J.C. (2018). Vitamin D in adults: Update on testing and supplementation. Geriatr. Psychol. Neuropsychiatr. Vieil..

[7] Hossein-nezhad A., Holick M.F. (2013). Vitamin D for health: A global perspective. Mayo Clin. Proc..

[8] Gil Á., Plaza-Diaz J., Mesa M.D. (2018). Vitamin D: Classic and novel actions. Ann. Nutr. Metab..

[9] Bayraktar N., Turan H., Bayraktar M., Ozturk A., Erdoğdu H. (2022). Analysis of serum cytokine and protective vitamin D levels in severe cases of COVID-19. J. Med. Virol..

[10] DiNicolantonio J.J., O’Keefe J.H. (2021). Magnesium and vitamin D deficiency as a potential cause of immune dysfunction, cytokine storm, and disseminated intravascular coagulation in COVID-19 patients. Mo. Med..

[11] Grant W.B., Lahore H., McDonnell S.L., Baggerly C.A., French C.B., Aliano J.L., Bhattoa H.P. (2020). Evidence that vitamin D supplementation could reduce risk of influenza and COVID-19 infections and deaths. Nutrients.

[12] Kong J., Zhu X., Shi Y., Liu T., Chen Y., Bhan I., Zhao Q., Thadhani R., Li Y.C. (2013). VDR attenuates acute lung injury by blocking Ang-2-Tie-2 pathway and renin-angiotensin system. Mol. Endocrinol..

[13] Yuan W., Pan W., Kong J., Zheng W., Szeto F.L., Wong K.E., Cohen R., Klopot A., Zhang Z., Li Y.C. (2007). 1,25- Dihydroxyvitamin D3 suppresses renin gene transcription by blocking the activity of the cyclic AMP response element in the renin gene promoter. J. Biol. Chem..

[14] Hoffmann M., Kleine-Weber H., Schroeder S., Krüger N., Herrler T., Erichsen S., Schiergens T.S., Herrler G., Wu N., Nitsche A. (2020). SARS-CoV-2 cell entry depends on ACE2 and TMPRSS2 and is blocked by a clinically proven protease inhibitor. Cell.

[15] Dijkman R., Jebbink M.F., Deijs M., Milewska A., Pyrc K., Buelow E., Van Der Bijl A., Van Der Hoek L. (2012). Replication-dependent downregulation of cellular angiotensin-converting enzyme 2 protein expression by human coronavirus NL63. J. Gen. Virol..

[16] Ji X., Zhang C., Zhai Y., Zhang Z., Zhang C., Xue Y., Tan G., Niu G. (2020). TWIRLS, an automated topic-wise inference method based on massive literature, suggests a possible mechanism via ACE2 for the pathological changes in the human host after coronavirus infection. bioRxiv.

[17] Chen I.Y., Chang S.C., Wu H.Y., Yu T.C., Wei W.C., Lin S., Chien C.-L., Chang M.-F. (2010). Upregulation of the chemokine (C-C motif) ligand 2 via a severe acute respiratory syndrome coronavirus spike-ACE2 signaling pathway. J. Virol..

[18] Yang J., Zhang H., Xu J. (2016). Effect of vitamin D on ACE2 and vitamin D receptor expression in rats with LPS-induced acute lung injury. Chin. J. Emerg. Med..

[19] Charoenngam N., Shirvani A., Holick M.F. (2021). Vitamin D and its potential benefit for the COVID-19 pandemic. Endocr. Pract..

[20] Meltzer D.O., Best T.J., Zhang H., Vokes T., Arora V.M., Solway J. (2021). Association of vitamin D levels, race/ethnicity, and clinical characteristics with COVID-19 test results. JAMA Netw. Open.

[21] World Medical Association (2013). Declaration of Helsinki: Ethical principles for medical research involving human subjects. JAMA.

[22] Serdar C.C., Cihan M., Yücel D., Serdar M.A. (2021). Sample size, power and effect size revisited: Simplified and practical approaches in pre-clinical, clinical and laboratory studies. Biochem. Med..

[23] Viechtbauer W., Smits L., Kotz D., Budé L., Spigt M., Serroyen J., Crutzen R. (2015). A simple formula for the calculation of sample size in pilot studies. J. Clin. Epidemiol..

[24] Sarhan R.M., Mohammad M.F., Boshra M.S. (2021). Differential clinical diagnosis and prevalence rate of allergic rhinitis, asthma and chronic obstructive pulmonary disease among COVID-19 patients. Int. J. Clin. Pract..

[25] Singh S., Nimavat N., Singh A.K., Ahmad S., Sinha N. (2021). Prevalence of Low Level of Vitamin D Among COVID-19 Patients and Associated Risk Factors in India–A Hospital-Based Study. Int. J. Gen. Med..

[26] Del Valle H.B., Yaktine A.L., Taylor C.L., Ross A.C. (2011). Dietary Reference Intakes for Calcium and Vitamin D..

[27] Biesalski H.K. (2020). Vitamin D deficiency and co-morbidities in COVID-19 patients–A fatal relationship?. Nfs J..

[28] Tan C.W., Ho L.P., Kalimuddin S., Cherng B.P.Z., Teh Y.E., Thien S.Y., Wong H.M., Tern P.J.W., Chandran M., Chay J.W.M. (2020). Cohort study to evaluate the effect of vitamin D, magnesium, and vitamin B12 in combination on progression to severe outcomes in older patients with coronavirus (COVID-19). Nutrition.

[29] Entrenas Castillo M., Entrenas Costa L.M., Vaquero Barrios J.M., Díaz J.F.A., Miranda J.L., Bouillon R., Gomez J.M.Q. (2020). Effect of calcifediol treatment and best available therapy versus best available therapy on intensive care unit admission and mortality among patients hospitalized for COVID-19: A pilot randomized clinical study. J. Steroid. Biochem. Mol. Biol..

[30] Ling S.F., Broad E., Murphy R., Pappachan J.M., Pardesi-Newton S., Kong M.F., Jude E.B. (2020). High-dose cholecalciferol booster therapy is associated with a reduced risk of mortality in patients with COVID-19: A cross-sectional multi-centre observational study. Nutrients.

[31] Barrea L., Gennari L., Merlotti D., Mingiano C., Frosali A., Giovanelli L., Torlasco C., Pengo M.F., Heilbron F., Soranna D. (2022). Vitamin D: A Role Also in Long COVID-19?. Nutrients.

[32] Campi I., Gennari L., Merlotti D., Mingiano C., Frosali A., Giovanelli L., Torlasco C., Pengo M.F., Heilbron F., Soranna D. (2021). Vitamin D and COVID-19 severity and related mortality: A prospective study in Italy. BMC Infect. Dis..

[33] Jain A., Chaurasia R., Sengar N.S., Singh M., Mahor S., Narain S. (2020). Analysis of vitamin D level among asymptomatic and critically ill COVID-19 patients and its correlation with inflammatory markers. Sci. Rep..

[34] Kaufman H.W., Niles J.K., Kroll M.H., Bi C., Holick M.F. (2020). SARS-CoV-2 positivity rates associated with circulating 25-hydroxyvitamin D levels. PLoS ONE.

[35] Ghasemian R., Shamshirian A., Heydari K., Malekan M., Alizadeh-Navaei R., Ebrahimzadeh M.A., Warkiani M.E., Jafarpour H., Bazaz S.R., Shahmirzadi A.R. (2021). The role of vitamin D in the age of COVID-19: A systematic review and meta-analysis. Int. J. Clin. Pract..

[36] Dissanayake H.A., de Silva N.L., Sumanatilleke M., de Silva S.D.N., Gamage K.K.K., Dematapitiya C., Kuruppu D.C., Ranasinghe P., Pathmanathan S., Katulanda P. (2021). Prognostic and therapeutic role of vitamin D in COVID-19: Systematic review and meta-analysis. J. Clin. Endocrinol. Metab..

[37] Chen J., Mei K., Xie L., Yuan P., Ma J., Yu P., Zhu W., Zheng C., Liu X. (2021). Low vitamin D levels do not aggravate COVID-19 risk or death, and vitamin D supplementation does not improve outcomes in hospitalized patients with COVID-19: A meta-analysis and GRADE assessment of cohort studies and RCTs. Nutr. J..

[38] Annweiler C., Beaudenon M., Simon R., Guenet M., Otekpo M., Célarier T., Gautier J., GERIA-COVID Study Group (2021). Vitamin D supplementation prior to or during COVID-19 associated with better 3-month survival in geriatric patients: Extension phase of the GERIA-COVID study. J. Steroid Biochem. Mol. Biol..

[39] Murai I.H., Fernandes A.L., Sales L.P., Pinto A.J., Goessler K.F., Duran C.S., Silva C.B.R., Franco A.S., Macedo M.B., Dalmolin H.H.H. (2021). Effect of a single high dose of vitamin D3 on hospital length of stay in patients with moderate to severe COVID-19: A randomized clinical trial. JAMA.

[40] Rastogi A., Bhansali A., Khare N., Suri V., Yaddanapudi N., Sachdeva N., Malhotra P. (2022). Short term, high-dose vitamin D supplementation for COVID-19 disease: A randomised, placebo-controlled, study (SHADE study). Postgrad. Med. J..

[41] Remmelts H.H., van de Garde E.M., Meijvis S.C., Peelen E.L., Damoiseaux J.G., Grutters J.C., Biesma D.H., Bos W.J., Rijkers G.T. (2012). Addition of vitamin D status to prognostic scores improves the prediction of outcome in community-acquired pneumonia. Clin. Infect. Dis..

[42] Infante M., Buoso A., Pieri M., Lupisella S., Nuccetelli M., Bernardini S., Fabbri A., Iannetta M., Andreoni M., Colizzi V. (2022). Low Vitamin D status at admission as a risk factor for poor survival in hospitalized patients with COVID-19: An Italian retrospective study. J. Am. Coll. Nutr..

[43] Lakkireddy M., Gadiga S.G., Malathi R.D., Karra M.L., Raju I.S.S.V., Chinapaka S., Baba K.S.S.S., Kandakatla M. (2021). Impact of daily high dose oral vitamin D therapy on the inflammatory markers in patients with COVID 19 disease. Sci. Rep..

[44] Annweiler C., Beaudenon M., Gautier J., Simon R., Dubée V., Gonsard J., Parot-Schinkel E., COVIT-TRIAL Study Group (2020). COvid-19 and high-dose VITamin D supplementation TRIAL in high-risk older patients (COVIT-TRIAL): Study protocol for a randomized controlled trial. Trials.

[45] Mariani J., Tajer C., Antonietti L., Inserra F., Ferder L., Manucha W. (2021). High-dose vitamin D versus placebo to prevent complications in COVID-19 patients: A structured summary of a study protocol for a randomised controlled trial (CARED-TRIAL). Trials.

